# Varying levels of 6-keto-prostaglandin F1α and thromboxane B_2_ in serum and endothelialization and hyperplasia in small-diameter grafts seeded with CD34+ bone marrow cells in canines

**DOI:** 10.3892/etm.2014.1573

**Published:** 2014-02-21

**Authors:** WEISHUAI LIAN, HUAYI ZHANG, KUN WANG, JUNHAO JIANG, ZIJIE SU, ZHENHAI YU

**Affiliations:** 1 Department of Vascular Surgery, Zhongshan Hospital, Fudan University, Shanghai 200032, P.R. China; 2 Department of Vascular Surgery, The Second Affiliated Hospital of Jiaxing Medical College, Jiaxing, Zhejiang 314000, P.R. China; 3 Department of General Surgery, Qianfoshan Hospital, Shandong University, Jinan, Shandong 250014, P.R. China

**Keywords:** CD34+ cells, grafts, endothelialization, 6-keto-prostaglandin F1α, thromboxane B_2_

## Abstract

The aim of the present study was to investigate the serum levels of 6-keto-prostaglandin (PG)F1α and thromboxane (TX)B_2_, as well as the endothelialization and hyperplasia of polytetrafluoroethylene (PTFE) and Dacron prostheses seeded with CD34+ cells in medium-term observation. A total of 24 crossbred dogs were randomly distributed into PTFE or Dacron groups. CD34+ cells were isolated from bone marrow aspirate and collected using an immunomagnetic bead-based system. The PTFE or Dacron prostheses were implanted into the abdominal aortic artery and inferior vena cava of the dogs. In each group, 8 dogs were implanted with prostheses that had been seeded with CD34+ cells, while 4 dogs were implanted with prostheses that had been seeded with autogenous blood as a control. Serum concentrations of 6-keto-PGF1α and TXB_2_ were determined at days 0, 10, 30 and 60 following surgery. The grafts were removed and examined at days 10, 30, 60 and 100 following surgery. Finally, CD34 factor staining was used to identify endothelial cells, while light and electron microscopy were applied to examine endothelialization and patency. The results revealed that confluent endothelial cells appeared on the neointima of prostheses seeded with CD34+ cells at day 30 following surgery. In the control groups compared with the experimental groups, there were fewer endothelial cells and the neointima was significantly thicker in the arterial (PTFE, 174±1.41 vs. 117±2.83 μm, respectively; P=0.001; Dacron, 187.5±3.5 vs. 100±1.41 μm, respectively; P<0.001) and venous (PTFE, 230.5±6.36 vs. 135±5.66 μm, respectively; P=0.001; Dacron, 249±2.83 vs. 121.5±3.54 μm, respectively; P<0.001) prostheses. In the experimental groups, intimal hyperplasia in the venous prostheses (PTFE, 135±5.66 μm; Dacron, 121.5±3.54 μm) was more severe compared with that in the arterial prostheses (PTFE, 117±2.83 μm; Dacron, 100±1.41 μm) at day 60. Compared with the 6-keto-PGF1α concentrations in the experimental groups, those in the control groups were significantly lower on day 10 (PTFE, 135±6.01 vs. 80.5±4.35 pg/l, respectively; P=0.001; Dacron, 145±6.54 vs. 81.2±5.10 pg/l, respectively; P<0.001) and were then maintained at a lower level. By contrast, the TXB_2_ concentration, following marked increases on day 10 in the experimental and control groups (PTFE, 635±32.8 vs. 1,256±63.5 pg/l, respectively; P<0.001; Dacron, 652±30.9 vs. 1,136±53.2 pg/l, respectively; P=0.001), remained at a high level in the control groups. Therefore, the results of the present study indicate that it is possible to achieve rapid endothelialization in PTFE or Dacron prostheses by implanting CD34+ cells. Endothelialization inhibited the reduction in the concentration of 6-keto-PGF1α and the increase in the concentration of TXB_2_. In addition, endothelialization inhibited excessive intimal hyperplasia and thrombosis. Thus, CD34+ cell seeding provides a theoretical basis for the clinical application of artificial vessel endothelialization.

## Introduction

Prostheses have been used as substitutes for large-diameter (>6 mm) arteries with satisfactory results since the 1950s due to the successful development of silk, Dacron and polytetrafluoroethylene (PTFE) prostheses (1). However, to date, there are no prostheses that achieve good patency when used to replace small-diameter (≤6 mm) arteries or veins in vascular surgery. However, it is possible to achieve improved healing and function of vascular grafts with a proper seeding technique and an appropriate cell source (2).

Mature endothelial cells have been shown to circulate in fresh peripheral blood (3). These cells, detached from the lining of the cardiovascular tree, may be a source of fallout healing (4) to endothelialize the flow surfaces of grafts. Bone marrow contains pluripotent CD34+ cells, which are known to produce hematopoietic cells. Studies *in vitro* have shown that CD34+ cells can differentiate into mature endothelial cells (5,6). Endothelial cells are known to produce an antithrombogenic blood-compatible surface; thus, the development of an endothelial monolayer on the luminal surface of synthetic vascular grafts is likely to prevent thrombus formation and improve long-term patency rates.

Prostacyclin (PGI_2_) exerts its antithrombotic and vasodilator function and is formed from arachidonic acid that is present in cellular membranes. PGI_2_ is produced by sequential activities of cyclooxygenases (COX) and PGI_2_ synthase in healthy endothelial cells and exerts its function through a paracrine signaling cascade that involves G-protein-coupled PGI_2_ receptors on nearby platelets and endothelial cells (7). PGI_2_ is unstable with a half-life between 2 and 3 min and is decomposed rapidly to 6-keto-prostaglandin (PG) F1α. Thus, the concentration of 6-keto-PGF1α indirectly reflects the concentration of PGI_2_ (8).

In the cardiovascular system, thromboxane (TX) A_2_ is predominantly derived from platelet COX-1. The interaction with the G-protein-coupled TXA_2_ receptor elicits not only platelet aggregation and smooth muscle contraction, but also the expression of adhesion molecules and the adhesion and infiltration of monocytes/macrophages. TXA_2_ is in homeostatic balance with PGI_2_ in the circulatory system (9). TXA_2_ is also unstable with a half-life between 5 and 7 min, and decomposes rapidly to TXB_2_. Therefore, the concentration of TXB_2_ indirectly reflects the concentration of TXA_2_. In this study, we investigatd whether it is possible to achieve rapid endothelialization in PTFE or Dacron prostheses by implanting CD34+ cells and the association between intimal hyperplasia or thrombosis and the concentration of 6-keto-PGF1α and TXB2.

## Materials and methods

### Animals

A total of 24 healthy young mongrel dogs were randomly divided into PTFE and Dacron groups. In each group, 8 dogs were implanted with prostheses that had been seeded with CD34+ cells, while 4 dogs were implanted with prostheses that had been seeded with autogenous blood only, as a control. The study was approved by the Animal Care and Use Committee of Shandong University (Jinan, China).

### Bone marrow collection and CD34 cell isolation

Following intraperitoneal anesthesia with 30 mg/kg pentobarbital sodium, 15–20 ml bone marrow was aspirated from the posterior superior iliac spine and mixed with heparin at a dose of 10 IU/ml. The mononuclear cells underwent Ficoll separation (specific gravity, 1.077) and were then diluted with phosphate-buffered saline (PBS) to a concentration of 2×10^7^ cells/ml. Cells were incubated with anti-canine CD34 monoclonal antibodies (BD Biosciences, Franklin Lakes, NJ, USA) and then CD34+ beads. The cell bead mixture was passed through the immunomagnetic separation system (immunomagnetic beads; Miltenyi Biotec, Bergisch Gladbach, Germany) and then washed several times to remove nonspecifically bound cells. The solution was released from the magnet and the positively selected CD34+ cells were eluted from the system. The selected cells were assessed by Trypan blue staining. The CD34 + cells were mixed with 1 ml 1X phosphate buffer saline (PBS) + 2% horse serum buffer and stained by CD34-FITC antibody (130-081-001; Miltenyi Biotec) and then Fc antibody was blocked, finally the CD34+ cells were analyzed by flow cytometry. Finally, the CD34+ cells were maintained at 4°C overnight.

### Graft preparation

A solution of 0.8 ml plasma, separated from peripheral venous blood, and 0.1 ml CaCl_2_ (2.5%) was injected into the graft lumen to form a layer of fibrin coagulum in the graft inner wall. The lumen measured 6 mm in diameter and 3 cm in length. The graft was clipped and rotated for ~5 min to ensure an adequate spread. The solution was then removed from the lumen and a mixture of 0.5 ml CD34+ cells, 0.5 ml plasma and 0.1 ml CaCl_2_ (2.5%) was injected into the graft lumen with vascular clamps in place at each end of the graft. The graft was rotated for ~20 min and maintained in a sterile Petri dish at room temperature for implantation. Only 1 ml plasma separated from canine peripheral venous blood was injected into the control grafts with vascular clamps in place at each end of the graft.

### Graft implantation

**Figure 1:**
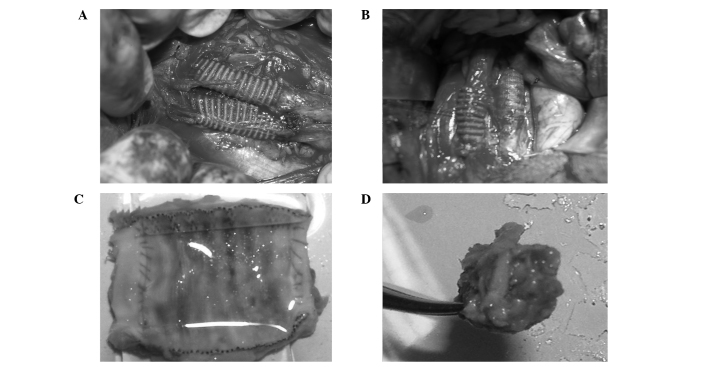
(A) Grafts were patent following surgery. (B) Grafts without significant deformation adjoined with the surrounding tissue. (C) In the test groups, grafts had smooth and translucent surfaces with no apparent thrombosis. (D) Venous grafts were occlusive in the control groups at day 100 following surgery.

In total, 24 mongrel dogs underwent surgery with intraperitoneal anesthesia of 30 mg/kg pentobarbital sodium. A midline laparotomy incision was performed and the infrarenal abdominal aorta was isolated by blunt and sharp dissection. Following proximal clamping below the renal arteries and distal clamping above the aorto-iliac bifurcation, a 2-cm segment of the abdominal aorta was resected. The grafts were then implanted with 5-0 Prolene sutures for end-to-end anastomosis. The same approach was performed on the inferior vena cava (Fig. 1A). Postoperatively, 100 mg/day antiplatelet drug (aspirin) was administered for 2 months.

### Blood collection and testing

Venous blood samples were collected during surgery and at days 10, 30 and 60 following surgery. Serum concentrations of 6-keto-PGF1α and TXB_2_ were determined by enzyme immunoassays. Kits for 6-keto-PGF1α (515211.1) and TXB_2_ (519031.1) were purchased from Cayman Chemical Co. (Ann Arbor, MI, USA).

### Specimen evaluation

At days 10, 30, 60 and 100 following surgery, 2 dogs from the PTFE experimental group and 2 dogs from the Dacron experimental group were sacrificed by exsanguination following the induction of deep anesthesia. At day 60 and 100, 2 dogs from the PTFE control group and 2 dogs from the Dacron control group were also sacrificed by exsanguination. The specimens were removed, opened longitudinally and photographic images were captured. Next, the tissue samples were evaluated with hematoxylin and eosin (H&E) staining, immunohistochemical analysis of factor VIII (FVIII) and CD34 antigen and scanning and transmission electron microscopy.

### H&E staining

The specimens were fixed in 10% formaldehyde solution and embedded in paraffin. Specimens were stained with hematoxylin for 5 min and flushed once with PBS. Hydrochloric acid and ethanol were used for separation and washed once with PBS. Eosin staining was performed for 3 min followed by PBS flushing. Finally specimens were dehydrated with alcohol and sealed with neutral gum seal.

### Immunohistochemical detection

Paraffin sections were placed into 3% H_2_O_2_-PBS at room temperature for 15 min, then washed with PBS three times every 3 min. The microwave antigen repair was performed as follows: slices were put into the repair of liquid (citrate buffer) for 10 min at 95°C and 20–30 min at the room temperature and washed with PBS three times every 3 min, then blocked by normal sheep serum at wet box of 37°C for 1 h or 4°C overnight. The sealing fluid was absorbed and primary antibodies were added at 4°C for the night. PBS wash was performed three times every 3 min, biotin-labeled secondary antibodies were added and reacted at 37°C for 30 min to 1 h. PBS wash was performed three times every 3 min, 35 to 50 μl horseradish peroxidase-labeled streptavidin was added and reacted at 37°C for 30 min to 1 h. PBS wash three times every 3 min, DAB was added and color development was performed without light (PBS 50 ml +20 mg DAB+7 μl 30% H_2_O_2_). Finally, slices were counterstained with 10% hematoxylin, washed with 1% hydrochloric acid-ethanol, dehydrated with alcohol and sealed with neutral gum.

### Scanning electron microscopy test

Specimens were flushed with heparin saline, fixed by glutaraldehyde and 1% osmic acid, dehydrated by ethanol, embedded with epoxy resin, double stained by uranyl acetate-lead citrate and then photographed with scanning electron microscopy.

### Statistical analysis

All experimental parameters were analyzed for statistical significance using SPSS software, version 16.0 (SPSS, Inc., Chicago, IL, USA). The Student’s t-test or one way analysis of variance was used to compare the statistical significance of the differences between the two groups. P<0.05 was considered to indicate a statistically significant difference.

## Results

### General observations

The grafts were patent following surgery (Fig. 1A). All the grafts were observed to adhere tightly to the surrounding tissue. There were no apparent deformations or signs of infection (Fig. 1B). In the experimental groups, the longitudinal section at day 10 showed the neointima covering part of the cavity surface, while at day 30 the neointima completely covered the graft and there was no thrombosis or stenosis present (Fig. 1C). Graft stenosis differed between day 60 and 100 (Table I) and the stenosis rates from the PTFE and Dacron test groups were significantly lower compared with those of the respective control groups at day 60 (P=0.001) and day 100 (P<0.001). All venous grafts were occlusive (Fig. 1D) in the control groups at day 100 following surgery. There was no indication of infarction in the lower limb or other organs in any of the 24 dogs.

### Identification of viable CD34+ cells

**Figure 2:**
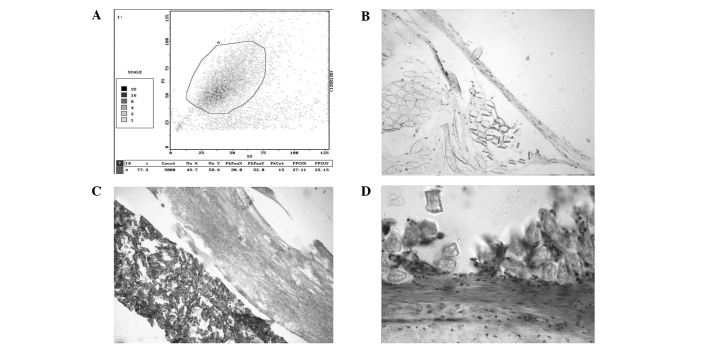
(A) CD34+ cells were identified by flow cytometry. (B) Immunocytochemical staining with FVIII/vWF and CD34 antibodies resulted in staining of endothelial cells. (C) With the PTFE test grafts, there was a layer of neointima consisting of a single layer of endothelial cells on the surface, shown to be positive with H&E staining. The subintima was largely composed of smooth muscle cells, fibroblasts and collagen. (D) With the Dacron test grafts, the subintima was largely composed of smooth muscle cells, fibroblasts and collagen. There were no osteocytes, osteoblasts or microcalcification in the seeded grafts. PTFE, polytetrafluoroethylene; H&E, hematoxylin and eosin; FVIII, factor VIII; vWF, von Willebrand factor.

Cells isolated by the immunomagnetic bead-based system were identified to be CD34+ cells by flow cytometry (Fig. 2A). The average number of viable CD34+ cells isolated in each group was 2.6±0.3×10^7^ cells/ml, as determined by Trypan blue exclusion prior to seeding.

### Identification of endothelial cells

Immunocytochemical staining with FVIII/von Willebrand factor and CD34 antibodies resulted in staining of endothelial cells (Fig. 2B). On the seeded grafts, there was a layer of neointima consisting of a single layer of endothelial cells on the surface, shown to be positive with H&E staining. There were varying amounts of fibrin coagulum with some blood cells (Fig. 2C). The subintima was largely composed of smooth muscle cells, fibroblasts and collagen. There were no osteocytes, osteoblasts or microcalcification in the seeded grafts (Fig. 2D).

### Calculation of neointimal thickness

**Figure 3:**
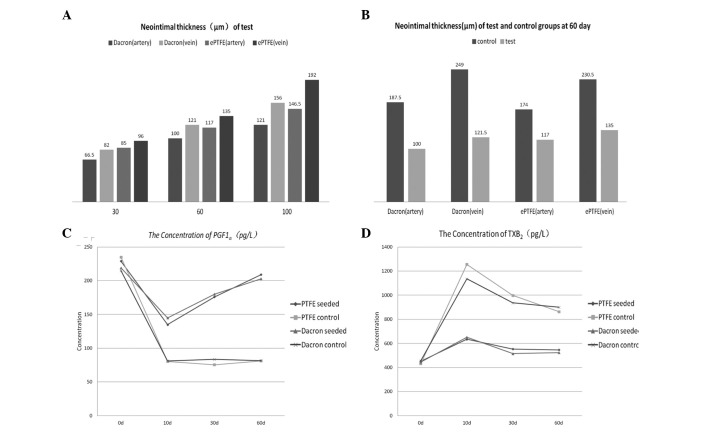
Endometrial thickness measurements of (A) the test groups and (B) the test and control groups at day 60. Serum concentration of (C) 6-keto-PGF1α and (D) TXB_2_. PG, prostaglandin; TX, thromboxane; PTFE, polytetrafluoroethylene.

Endometrial thickness measurements are shown in Fig. 3A and B. There were significant differences in neointimal thickness between the experimental and control groups (PTFE, artery P<0.001 and vein P<0.001; Dacron, artery P=0.001 and vein P<0.001; Fig. 3B). However, no significant differences were identified between the two CD34+ cell-seeded groups (P=0.84; Fig. 3A).

### Concentration of 6-keto-PGF1α and TXB_2_

Postoperatively, new endothelial cells in the experimental groups synthesized significantly higher levels of PGI_2_ compared with the levels in the control groups (PTFE, P=0.001; Dacron, P=0.001; Fig. 3C). In the control groups, vascular damage was not repaired as quickly, the platelet release reaction was enhanced and TX synthesis was significantly increased. By contrast, in each of the experimental groups, due to the protective effect of the endothelial cells, the TXB_2_ concentration was significantly lower compared with that in the respective control group (PTFE, P<0.001; Dacron, P=0.001; Fig. 3D).

### Scanning and transmission electron microscopy

**Figure 4:**
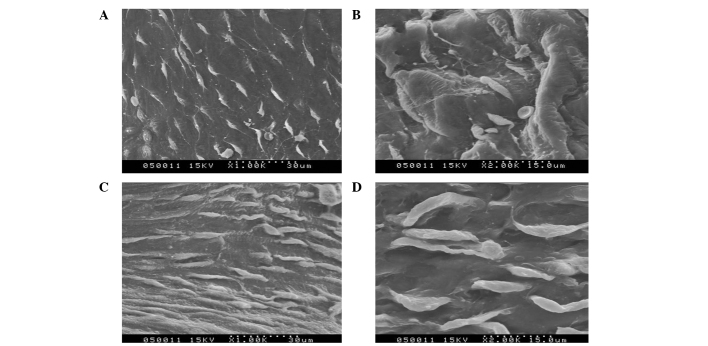
Scanning and transmission electron microscopy images of the (A) Dacron test group at day 10 (magnification, ×1,000), (B) PTFE test group at day 10 (magnification, ×2,000), (C) Dacron test group at day 30 (magnification, ×1,000) and (D) PTFE test group at day 30 (magnification, ×2,000). PTFE, polytetrafluoroethylene.

**Figure 5:**
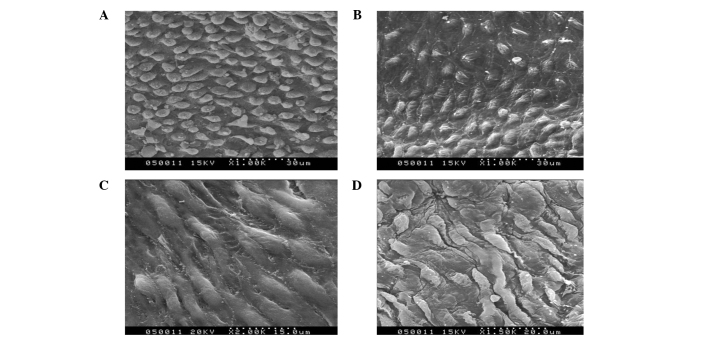
Scanning and transmission electron microscopy images of the (A) Dacron test group at day 60 (magnification, ×1,000), (B) PTFE test group at day 60 (magnification, ×1,000), (C) Dacron test group at day 60 (magnification, ×2,000) and (D) PTFE test group at day 60 (magnification, ×1,500). PTFE, polytetrafluoroethylene.

**Figure 6:**
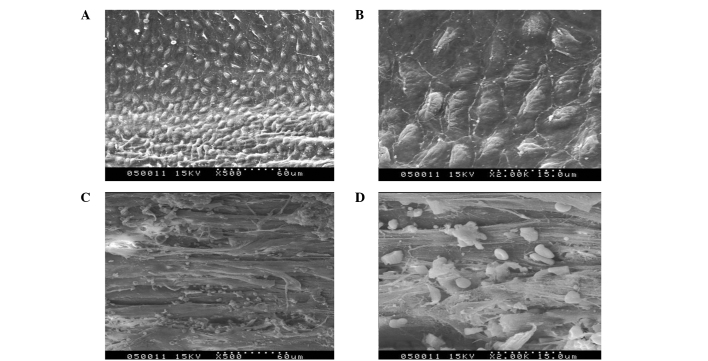
Scanning and transmission electron microscopy images of (A) Dacron test group at day 100 (magnification, ×500), (B) PTFE test group at day 100 (magnification, ×2,000), (C) Dacron control group at day 60 (magnification, ×500) and (D) PTFE control group at day 60 (magnification, ×2,000). PTFE, polytetrafluoroethylene.

In the experimental groups at day 10, the density of the endothelial cells was low and the cells exhibited spindle morphology. The transformation of specific endothelial cells into spindles was visible in the images of scanning and transmission electron microscopy (Fig. 4A and B). In the experimental groups at day 30, a large number of endothelial cells appeared at the anastomoses. In addition, the number of endothelial cells gradually decreased from the ends to the middle of the graft (Fig. 4C and D). At day 60, the scanning and transmission electron microscopy images showed a continuous layer of endothelial cells on the CD34+ cell-seeded graft surfaces. Additionally, the closer the anastomoses, the higher the number of endothelial cells (Fig. 5A and B). In the middle of the grafts, endothelial cells were sparse but almost covered the surface (Fig. 5C and D). In the experimental groups at day 100, endothelial cells were arranged tightly and were more mature (Fig. 6A). The endothelial cells were arranged more closely at the anastomoses than elsewhere. At high magnification, it was evident that the cells were spread well with rich pseudopodia (Fig. 6B).

On the control grafts, the majority of the surface was covered with a film of fibrin material mixed with blood cells. In addition, a small amount of necrosis and senescence was present in the endothelial cells at day 60 (Fig. 6C and D).

## Discussion

Vessel prostheses have been widely used in vascular surgery; however, small-caliber artificial blood vessels have not produced satisfactory results. Bypass graft failure, as a result of acute thrombosis and intimal hyperplasia, has been the major challenge for surgical procedures involving small-diameter vascular prostheses (10). A lack of endothelial cells in the inner surface of the prostheses is the main reason for the lower patency rates (11).

In 1984, Civin *et al* (12) identified CD34+ cells for the first time. CD34+ cells include pluripotent hematopoietic progenitor cells and are defined by the expression of their surface antigen, which is a mucin-like cell surface glycoprotein (13). Previous studies (14,15) with human peripheral blood and umbilical cord blood support the derivation of circulating outgrowth endothelial cells from a small subset of CD34+ cells.

In previous years, endothelialization with CD34+ cells seeded in artificial vasculature has shown enormous potential. However, the experiments used short experimental times and *in vitro* cultures, which may be affected by a number of factors; thus, the procedure is yet to be applied in clinical practice (16). Based on these circumstances, the present study investigated the medium-term results of CD34+ cell seeding in small-diameter artificial vessels.

In the present study, endothelialization and stenosis were investigated in prostheses seeded with CD34+ cells. Confluent endothelial cells appeared on the neointima of prostheses seeded with CD34+ cells, as shown by light and electron microscopy. In addition, there were fewer endothelial cells and a film of fibrin material mixed with blood cells in the control group. Therefore, the results indicate that pre-seeding with CD34+ cells in vascular prostheses may result in rapid endothelialization and prevent platelet aggregation and thrombus formation effectively.

Scanning electron microscopy observations revealed that the PTFE grafts had a less complete neointima compared with that on the Dacron grafts, as cellulose and blood cells were attached to the surface. In addition, following H&E staining, the PTFE neointima was shown to separate partly from the vascular surface and the cell density was lower compared with that in the Dacron experimental group. This may be associated with the structure of the Dacron artificial blood vessels, as the inner surface is relatively rough compared with that of the PTFE grafts.

Serum concentrations of 6-keto-PGF1α and TXB_2_ following surgery were determined. In the control groups, the 6-keto-PGF1α concentrations decreased significantly (P=0.01) and were then maintained at a lower level. By contrast, the 6-keto-PGF1α concentrations in the experimental groups were significantly higher compared with those in the control groups. This may be due to the neovascular endothelial cells synthesizing more PGI_2_, which is unstable and decomposes rapidly to 6-keto-PGF1α.

The TXB_2_ concentration exhibited a marked increase (P=0.01) that remained at a high level, which indicated that platelets were activated and synthesizing greater quantities of TXA_2_. Higher TXB_2_ levels were observed in the control groups than in the experimental groups. This may be due to: i) the missing intima induced activated platelets to produce more TXA_2_. ii) the protective effect of the neointima inhibited platelet adhesion and activation; and/or iii) high PGI_2_ levels may have antagonized the production of TXB_2_ in the experimental group.

In the present study, artificial vessel endothelialization was shown to be viable with CD34+ cell seeding. In addition, the procedure inhibited excessive reductions in the concentration of 6-keto-PGF1α and increases in the concentrations of TXB_2_. Excessive intimal hyperplasia and thrombosis were also inhibited. Improved stenosis rates were obtained in the medium-term, which provides a theoretical basis for the clinical application of artificial vessel endothelialization.

## Figures and Tables

**Table I:** Graft stenosis rate following surgery, %.

	Day 60				Day 100			
		
	Test		Control		Test		Control	
				
Site	PTFE	Dacron	PTFE	Dacron	PTFE	Dacron	PTFE	Dacron
A	0, 9	0, 18	36, 48	35, 52	17, 43	12, 64	65, 81	82, 91
V	27, 41	38, 45	67, 83	75, 78	78, 100	87, 100	100, 100	100, 100

PTFE, polytetrafluoroethylene; A, arterial; V, venous. Values are for the two animals in each group evaluated at each time point.
